# PhiSiGns: an online tool to identify signature genes in phages and design PCR primers for examining phage diversity

**DOI:** 10.1186/1471-2105-13-37

**Published:** 2012-03-04

**Authors:** Bhakti Dwivedi, Robert Schmieder, Dawn B Goldsmith, Robert A Edwards, Mya Breitbart

**Affiliations:** 1 College of Marine Science, University of South Florida, St. Petersburg FL 33701, USA; 2 Department of Computer Sciences and Biology, San Diego State University, San Diego CA 92182, USA; 3 Computational Science Research Center, San Diego State University, San Diego CA 92182, USA; 4 Mathematics and Computer Science Division, Argonne National Laboratory, Argonne IL 60439, USA

## Abstract

**Background:**

Phages (viruses that infect bacteria) have gained significant attention because of their abundance, diversity and important ecological roles. However, the lack of a universal gene shared by all phages presents a challenge for phage identification and characterization, especially in environmental samples where it is difficult to culture phage-host systems. Homologous conserved genes (or "signature genes") present in groups of closely-related phages can be used to explore phage diversity and define evolutionary relationships amongst these phages. Bioinformatic approaches are needed to identify candidate signature genes and design PCR primers to amplify those genes from environmental samples; however, there is currently no existing computational tool that biologists can use for this purpose.

**Results:**

Here we present PhiSiGns, a web-based and standalone application that performs a pairwise comparison of each gene present in user-selected phage genomes, identifies signature genes, generates alignments of these genes, and designs potential PCR primer pairs. PhiSiGns is available at (http://www.phantome.org/phisigns/; http://phisigns.sourceforge.net/) with a link to the source code. Here we describe the specifications of PhiSiGns and demonstrate its application with a case study.

**Conclusions:**

PhiSiGns provides phage biologists with a user-friendly tool to identify signature genes and design PCR primers to amplify related genes from uncultured phages in environmental samples. This bioinformatics tool will facilitate the development of novel signature genes for use as molecular markers in studies of phage diversity, phylogeny, and evolution.

## Background

Phages (viruses that infect bacteria) are ubiquitous on Earth, where they are the most abundant and diverse biological entities [1-3]. Phages have been central to many tools and discoveries in molecular biology, and serve important ecological functions, including structuring microbial communities [4,5], driving evolution through gene transfer [6,7], and playing major roles in biogeochemical cycling [8,9]. Since phages are often host-specific predators [10,11], it is important to understand not only the abundance of phages, but also which types of phages are present in the environment.

Phages are extremely diverse, encompassing a wide range of virion properties, genome sizes and types, host ranges, and lifestyles. Phages are typically classified by the International Committee on Taxonomy of Viruses (ICTV) based on morphology and nucleic acid type [12] or by sequence-based taxonomic systems [13-16]. Traditional culture-based methods for exploring the diversity of phages in the environment are limited because they require having the bacterial host in culture, and it is known that the majority of environmental bacteria cannot be cultured using standard laboratory techniques [17,18]. Recently, molecular techniques have overcome these limitations, revealing a vast diversity of phages in natural environments without the requirement of culturing [19-23].

Development of the 16S ribosomal RNA gene as a molecular marker for studying microbial communities revolutionized the field of microbial ecology by allowing researchers to access the vast diversity of uncultured microbes in natural systems [24-26]. However, exploration and comparative genomics of environmental phage communities have been hampered by the lack of a universally conserved genetic marker that can be used to examine the diversity of phages and trace their evolutionary histories. Despite the fact that there is no single gene found in all known phages, groups of related phage genomes often share conserved genes ("signature genes") which have been used to examine phage diversity. For example, conserved regions of phage structural proteins, such as the portal vertex protein (*g20*) and the major capsid protein (*g23*), are routinely used to characterize genetic diversity in T4-like myophage communities [22,27-33]. Other studies have used the DNA polymerase gene for examining the diversity and evolution of T7-like podophages [20,21,23,34]. Numerous auxiliary metabolic genes (i.e., phage-encoded metabolic genes that were previously thought to be restricted to cellular genomes [2]) involved in photosynthesis, carbon metabolism, and nucleotide metabolism have also been used as signature genes for marine phages [30,35-39]. Although these signature genes are restricted to specific subsets of phage genomes and are not universally present in all phage types, they are good targets to design PCR primers for exploring related uncultured phages in environmental samples. Further examination of environmental phage diversity would be greatly enhanced through the development of PCR assays for additional signature genes.

With advances in sequencing technologies and the success of student-driven research/outreach programs [40], an increasing number of phage genomes are sequenced each year and are available for bioinformatic analyses [3]. As of February 2011, the genomes of 636 phages and 33 archaeal viruses were available in the PhAnToMe database (http://www.phantome.org/) [41]. Many phage ecologists are interested in mining these genomes to identify and design PCR primers for signature genes. Numerous tools and databases exist to identify and analyze homologous gene sequences (e.g., COGs [42], OrthoMCL [43], HMMER [44]). One major limitation of these existing tools is that they are confined to cellular organisms, and very few available tools incorporate viral genomes (e.g., CoreGenes [45], CoreExtractor [15]). Likewise, numerous tools for primer design and analysis exist (e.g., CODEHOP [46], IDT Oligo Analyzer [47], Primer3 [48]), yet they have many restrictions regarding input file requirements (based upon nucleotide sequence, protein sequence or multiple nucleotide alignment), primer type (non-degenerate or degenerate), genomes of interest, physicochemical properties, input and output format, and usability. In practice, the identification of conserved genes and design of PCR primers to amplify these genes currently requires several stand-alone steps that are not integrated into a single work flow. When performed manually, it can be a time-consuming, tedious, and error-prone process.

In light of these problems, PhiSiGns provides a convenient web interface that allows biologists to perform a dynamic search against selected phage genomes of interest, identify signature genes, generate sequence alignments, and design primers for PCR amplification, all in one environment that increases efficiency and productivity. Signature genes identified using this tool can be used to build phylogenetic trees and study phage evolution. Furthermore, primers designed using PhiSiGns can be used to amplify related sequences from environmental samples to increase knowledge of uncultured phage diversity.

## Methods

### Implementation

PhiSiGns was written in Perl 5.8 [49] and is available in both standalone and web-based versions (http://www.phantome.org/phisigns/; http://phisigns.sourceforge.net/). The web interface is implemented in Perl using the Common Gateway Interface (CGI) module to generate dynamic HTML content. The web version is currently running on a PC server with Fedora Linux using an Apache HTTP server to support the web services. The source code and documentation are freely available at http://phisigns.sourceforge.net/.

**Figure 1:**
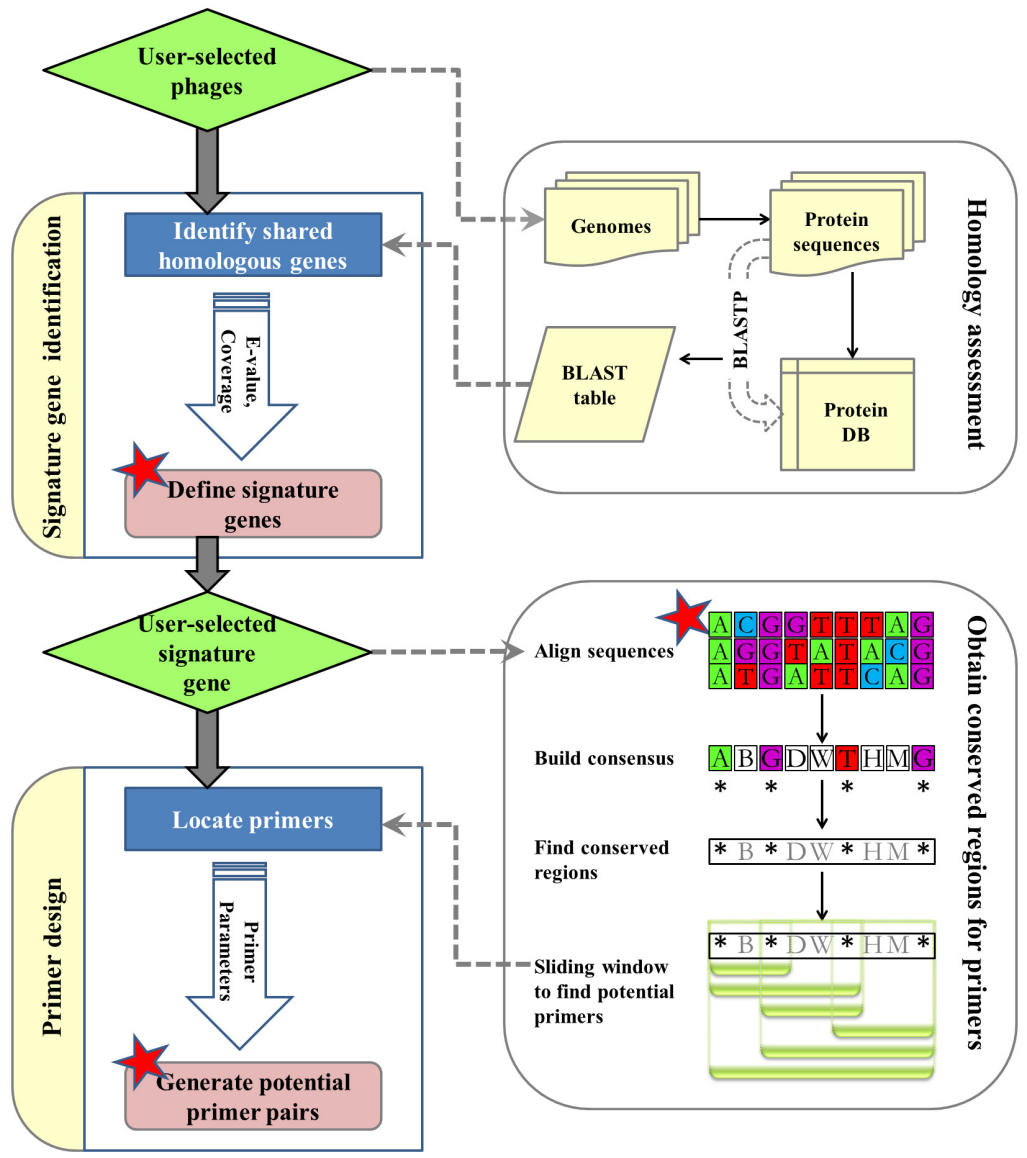
**An outline of the PhiSiGns workflow**. PhiSiGns consists of two critical, interlinked processes: 1) the identification of signature genes conserved amongst a group of phages, and 2) the design of PCR primers for the amplification of these signature genes. Red stars indicate results that can be downloaded to a local machine

PhiSiGns is an automated tool that runs pairwise comparisons of all the genes from user-selected phage genomes, identifies signature genes, generates sequence alignments, and designs primer pairs for PCR amplification (Figure 1). The tool begins with users selecting phages of interest from the list of available genomes in the phage genomic database (downloaded from PhAnToMe [41] in February 2011). Potential signature genes are identified based on pre-calculated BLASTP [50,51] pairwise sequence similarity search results. The phage database and pre-calculated BLAST outputs are updated annually. Subsequently, users can design primers for a selected signature gene using their preferred parameters. An alignment of the selected signature gene is generated using CLUSTALW (currently version 2.0.10) [52] with default settings, although users can choose to upload their own manually-curated alignment of the signature gene instead. From the nucleotide sequence alignment, a consensus sequence is built using the IUPAC ambiguity code. Conserved regions are extracted from the consensus sequence and used as a template to generate primers using a sliding window approach. Each unique primer sequence is tested for user-specified properties such as primer length, product size (target length to be amplified), degeneracy (computed by multiplying the degeneracy of each contributing IUPAC mixed base), GC content (the number of G's and C's in the primer as a percentage of the total bases), GC clamp (the presence of G's or C's within the last five bases from the 3' end of primers; there should be no more than three), maximum 3' stability (the maximum stability for the five 3' bases of a forward and reverse primer measured in ΔG; primers with ΔG ≥ -9 kcal/mol are considered acceptable for primer pairing), and melting temperature (temperature at which one half of the DNA duplex will dissociate and become single stranded). A primer complementarity test is also performed as part of the primer design process, including a check for self-dimers (intermolecular base pairing between sense primers or antisense primers), cross-dimers (intermolecular base pairing between the sense and antisense primers), and hairpin formation (intramolecular base pairing within sense primers or antisense primers). Primer pairs that meet user-specified parameters are output as potential primer pairs for the selected signature gene. All result files generated during the process (including the list of signature genes for the phages of interest, signature gene FASTA sequences, signature gene sequence alignment, and list of potential primer pairs) are displayed on the web page and directly downloadable.

### Case study: using PhiSiGns to design primers to examine the diversity of T7-like phages in sewage

Raw sewage samples were collected in February 2009 from a wastewater treatment facility in Manatee County, Florida. Virus particles were purified from 1.2 liters of sample by filtering through 0.45 μm and 0.2 μm Sterivex filters (Millipore, Billerica, MA, USA). Virus particles were further concentrated and purified using PEG precipitation followed by CsCl gradient centrifugation [53]. Viral DNA was extracted using the MinElute Virus Spin Kit (Qiagen, Valencia, CA, USA).

PhiSiGns was used to identify signature genes amongst the eight completely sequenced "core" T7-like phage genomes (Enterobacteria phage T7, Enterobacteria phage T3, Enterobacteria phage K1F, *Yersinia pestis *phage phiA1122, *Yersinia *phage Berlin, *Yersinia *phage phiYeO3-12, *Vibrio *phage VP4, and *Pseudomonas *phage gh-1) as proposed by Lavigne et al. (2008). Forward (5'-ACHGARGGYGARATHG-3') and reverse (5'-CVCCTTGYTGRTTDC-3') primers were designed using PhiSiGns to amplify a ~ 838 bp region of the primase/helicase gene from T7-like phages. The 50 μL PCR mixture contained 2 U Apex Taq DNA Polymerase (Genesee Scientific, San Diego, CA), 1× Apex Taq Reaction Buffer, 2 mM Apex MgCl2, 1 μM each primer, 0.2 mM dNTPs, and 4 μL of template DNA. The reaction conditions were (i) 5 minutes of initial denaturation at 94°C; (ii) 30 cycles of (a) one minute of denaturation (94°C), (b) one minute of annealing (51.1°C - 0.5°C/cycle), (c) two minutes of extension (72°C); and (iii) 10 minutes of final extension at 72°C. After amplification, the PCR product was cleaned with the MoBio UltraClean PCR Clean-Up Kit (MO BIO Laboratories, Carlsbad, CA) and cloned using the TOPO TA Cloning Kit for Sequencing (Invitrogen, Carlsbad, CA). Positive transformants were sequenced by Beckman Coulter Genomics (Danvers, MA). Vector and low-quality sequences were trimmed with Sequencher 4.7 (Gene Codes, Ann Arbor, MI) and sequences were compared against the NCBI non-redundant database using BLASTX to identify sequences with similarity to the primase/helicase of T7-like phages.

The T7-like primase/helicase sequences were de-replicated using FastGroupII [54] by considering sequences with ≥ 99% nucleotide identity as identical. The 50 unique sequences recovered from the sewage samples [GenBank: JN180326-JN180375] were aligned with T7-like phages from GenBank using CLUSTALW [52] as implemented in MEGA v5.0 [55]. A phylogenetic tree was then constructed on the aligned dataset using PhyML v3.0 [56]. Maximum-likelihood analysis was performed using the GTR nucleotide substitution model with six substitution rate categories and parameters (base frequencies, proportion of invariable sites, gamma distribution) estimated from the dataset. One thousand bootstrap replicates were performed to assess statistical support for the tree topology.

## Results

PhiSiGns provides a single web interface that combines two essential processes: (i) the identification of signature genes sharing amino acid sequence similarity; and (ii) the design of PCR primers (degenerate or non-degenerate) for the amplification of these signature genes.

### Identification of signature genes

**Figure 2:**
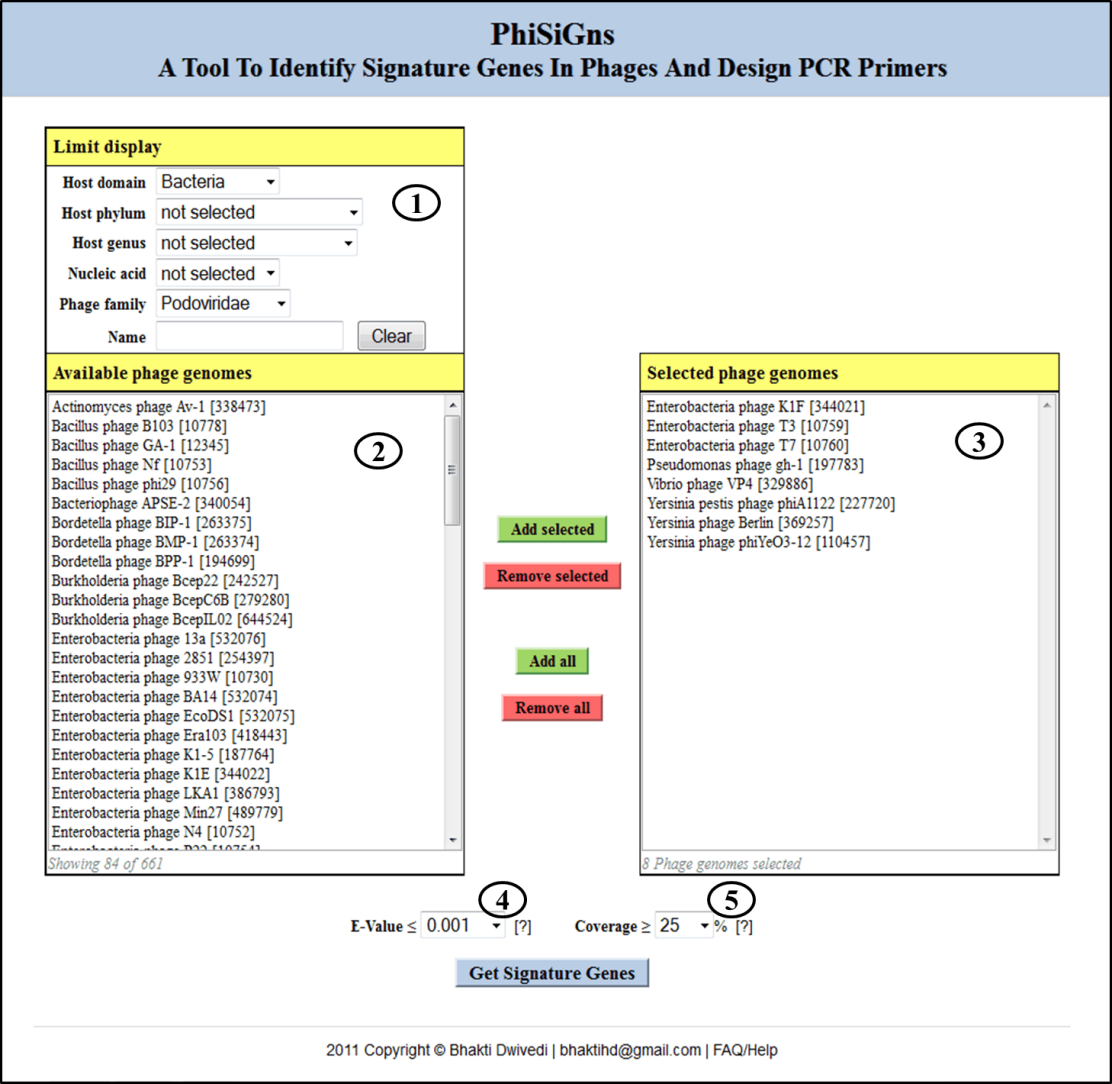
**Web interface for the identification of signature genes**. The interface shows 1) the options to limit the display of available phage genomes, 2) a list of phage genomes meeting the selection criteria, 3) a list of the user-selected phage genomes from step (2) for signature gene identification and primer design, 4) the BLAST E-value cut-off for similarity searches, and 5) the BLAST alignment coverage cut-off for similarity searches. Users can click on the question marks for additional information on a given parameter

For signature gene identification, users select phages of interest from the list of completely sequenced phage genomes (Figure 2 displays this user interface). This dataset is derived from the phage database on the PhAnToMe website [41] and can be sorted using different classification criteria such as phage name, nucleic acid type, phage family, host domain, host phylum, and host genus. Gene annotations for the protein coding regions are imported from the SEED [57,58] and the records that lack annotation are extracted from GenBank. Once the phages are selected, all protein sequences from each phage genome are screened using a BLASTP sequence similarity search [50,51] to determine if a homologous protein exists in any of the other selected phage genomes. These all-against-all protein pairwise comparisons are pre-calculated using the BLASTP program implemented in the BLAST stand-alone package [59] with a default E-value cut-off of 10. The best hits for each protein amongst the selected genomes are retrieved from these pre-calculated BLAST results based on a user-defined E-value and alignment coverage percentage (computed as alignment length divided by the average query and subject sequence length). Finally, the genes that are shared amongst multiple phage genomes are integrated into signature gene groups. The list of identified signature genes can be exported from the web browser as a tab delimited file.

### Primer design

**Figure 3:**
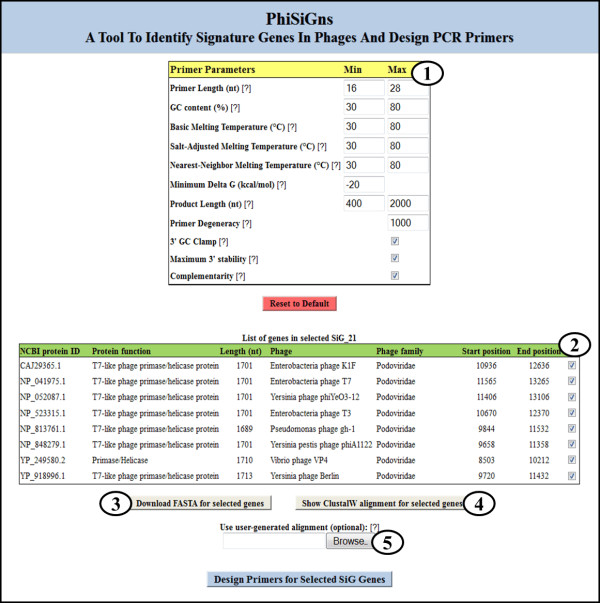
**Web interface for designing primers on a selected signature gene**. The interface shows 1) an input box for minimum and maximum values for the primer parameters, 2) a list of genes within the selected signature gene group (users have the option to select/deselect genes from the table for alignment and primer design), 3) an option to download the sequence FASTA file, 4) an option to view the program-generated CLUSTALW alignment, and 5) an option to upload a user-generated alignment for the selected signature gene, to be used for primer design

The PhiSiGns primer design web interface (Figure 3) allows users to design PCR primers (degenerate or non-degenerate) for a selected signature gene based on the multiple sequence alignment. In the first step, the nucleotide sequences for a given signature gene are translated and the protein sequences are aligned using the CLUSTALW [52] multiple sequence alignment program with default settings. Once the sequences are aligned, they are reverse-translated to their corresponding nucleotide sequences for subsequent steps. PhiSiGns also provides users with the option to upload their own nucleotide alignment of a selected signature gene for primer design. From the gene sequence alignment, an IUPAC consensus is computed with a 100% identity threshold and all potential conserved regions are then extracted from the consensus. A conserved region is defined as a region with a minimum of two completely conserved nucleotide bases (i.e., A, C, G or T; no mixed bases) within 19 bases of each other. Starting with the first conserved base, the program screens the next 19 bases to find another conserved base. If none are located, the program moves on to the next conserved base and begins the search again. If additional conserved bases are located within 19 bases of the original residue, the region between the furthest two of these bases is extracted along with an additional 5 bases upstream and downstream. These steps are repeated for each completely conserved base, and all regions containing gaps are excluded from the analysis. This process results in sequences between 12 and 30 bases in length that contain conserved bases and are sufficiently large to allow different primer design possibilities. For each conserved region identified, sequences ranging from 10-28 bases (the default range for the primer length) are obtained by a sliding window approach, moving one base at a time within the region. The length, start position, and stop position of each potential primer are recorded.

Potential primer sequences are then analyzed for several physicochemical properties including primer length, primer degeneracy, product size, GC content, GC clamp, melting temperature, maximum 3' stability, and complementarity (self-dimer, cross-dimer, and hairpin formation). The equations and values used in all thermodynamic calculations are available at http://phisigns.sourceforge.net/. Melting temperatures (T_m_) are calculated using three different methods: (1) basic melting temperature [60,61], (2) salt-adjusted melting temperature [62,63], and (3) nearest-neighbor melting temperature [64]. The Gibbs free energy (ΔG kcal/mol) is computed to measure the minimum ΔG and maximum 3' end stability of a primer sequence. ΔG is the measure of the spontaneity of the reaction, representing the energy required to break the secondary structure. Larger negative values for ΔG indicate more self-priming and stable, undesirable secondary structures. Primer pairs are also tested for self-dimers, cross-dimers and hairpins. Primer-dimers and hairpins must have less than five consecutive base pairings to be considered as potential primer pairs. The user can input the desired minimum and maximum properties for each of these primer parameters, or rely on the default parameters provided (Figure 3). PCR primers for amplifying target regions are then paired by minimizing differences in melting temperature between the forward and reverse primers, while conforming to the user-desired primer and product parameters.

### Case study: using PhiSiGns to design primers to examine the diversity of T7-like phages in sewage

T7-like phages are short-tailed, double-stranded DNA phages with genomes of ~40 kb in length, belonging to the *Podoviridae *family [12]. The abundance and high genetic diversity of T7-like podophages have been previously documented using highly conserved genes such as the DNA polymerase in a wide range of environments [15,20,21,34,65]. To demonstrate the utility of PhiSiGns, the program was used to identify signature genes amongst the eight completely sequenced "core" T7-like phage genomes (Enterobacteria phage T7, Enterobacteria phage T3, Enterobacteria phage K1F, *Yersinia pestis *phage phiA1122, *Yersinia *phage Berlin, *Yersinia *phage phiYeO3-12, *Vibrio *phage VP4, *and Pseudomonas *phage gh-1) as proposed by Lavigne et al. (2008). Using an E-value cut-off of 0.001 and 10 percent alignment coverage cut-off, PhiSiGns identified 58 signature genes conserved amongst members of this group (Table 1). Of these 58 signature genes, 24 are present in all eight genomes, including genes for replication (e.g., DNA polymerase, DNA-directed RNA polymerase, primase/helicase, single-stranded DNA binding protein, DNA ligase), packaging (e.g., DNA packaging protein A, exonuclease, endonuclease, terminase, RNA polymerase inhibitor), structural proteins (e.g., phage capsid and scaffold, portal connector protein, tail fiber, internal core proteins), cell lysis proteins (e.g., holins, lysins), and a few unknown phage proteins.

**Table 1:** Overview of signature genes (SiGs) identified amongst eight core T7-like phage genomes in the PhiSiGns case study

# of phages	# of SiGs	Functional roles
8	24	DNA polymerase, RNA polymerase, primase/helicase, ssDNA binding protein, ligase, packaging protein A, exonuclease, endonuclease, terminase, RNA polymerase inhibitor, phage capsid and scaffold, portal connector protein, tail fiber, internal core proteins, holins, lysins, unknown phage proteins

7	3	endopeptidases, unknown phage proteins

6	6	kinase, ssDNA binding protein, dGTPase, unknown phage proteins

5	6	nuclease, lipoprotein, unknown phage proteins

4	3	primase/helicase, unknown phage proteins

3	4	adenosylmethionine hydrolase, unknown phage proteins

2	12	endonuclease, unknown phage proteins

**Figure 4:**
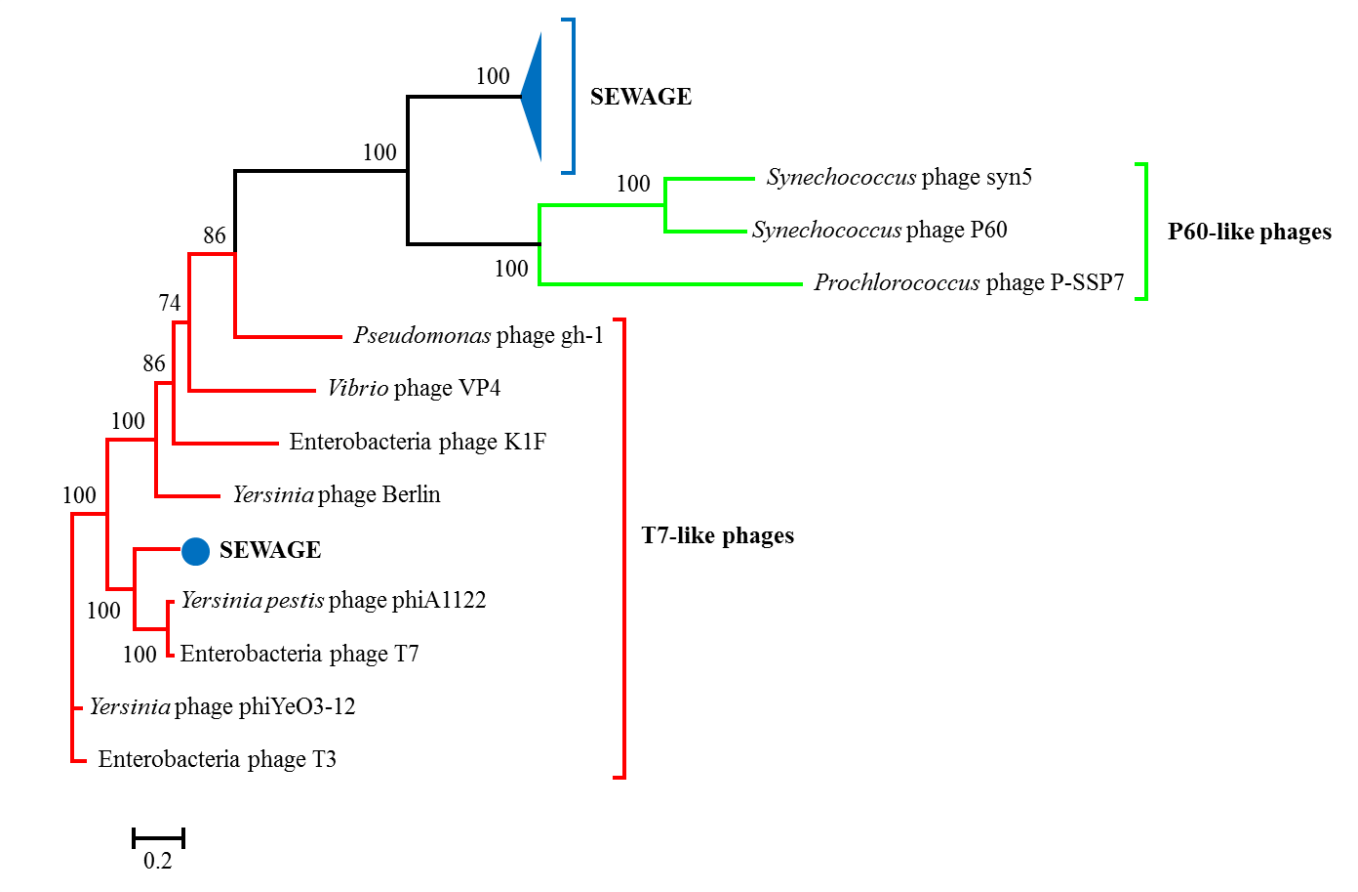
**Phylogenetic tree of T7-like primase/helicase sequences amplified from sewage samples with degenerate primers designed with PhiSiGns**. The eight core T7-like phages and three cyanophage P60-like phages are shown in red and green, respectively. The sewage sequences amplified in this study are shown in blue. The SEWAGE clade represents the compressed view of 49 closely related sequences recovered from sewage in this study. Internal nodes with bootstrap support ≥ 70% are shown with the corresponding bootstrap value indicated. The scale bar represents the number of nucleotide substitutions per site.

For this case study, the primase/helicase gene was chosen for the design of degenerate PCR primers to demonstrate the utility of PhiSiGns and explore the diversity of T7-like phages in raw sewage samples (see methods section). A total of 96 sequences were obtained from the sewage samples, 62 of which had best hits to phage primase/helicase proteins based on BLASTX against the GenBank non-redundant database confined to viruses. The 62 T7-like primase/helicase sequences were then de-replicated with FastGroupII [54] by considering sequences with ≥ 99% identity at the nucleotide level as identical, resulting in 50 unique sequences. A phylogenetic tree was then constructed using these uncultured phage sequences from sewage along with primase/helicase sequences from the cultured core T7-like phages and several P60-like cyanophages (Figure 4). Almost all of the sequences amplified using the PhiSiGns primers are very closely related to each other and form a clade (designated as "SEWAGE" in the tree) that is distinct from the cultured T7-like phages. In addition, the SEWAGE clade forms a sister group with the P60-like cyanophages, suggesting that the primase/helicase of these sewage phages may be more closely related to the cyanophages than to the core T7-like phages. However, one sewage sequence falls within the clade of core T7-like phages, closely grouping with Enterobacteria phage T7 and *Yersinia pestis *phage phiA1122.

## Discussion

To understand phage evolution and ecology, it is crucial to identify common genes that share sequence similarity in different phages and can be used for phylogenetic comparisons. PhiSiGns provides a simple, user-friendly platform to enable phage biologists to identify signature genes and then design primers for PCR amplification of related sequences from uncultured environmental phage communities. PhiSiGns can be applied to examine any user-specified group of phages, such as phages that infect a common host, phages that were originally isolated from the same environment, or phages with a certain ICTV classification. Comparison of signature gene sequences from cultured phages and those amplified from environmental samples using primers designed with PhiSiGns can yield insight into phage diversity and evolution.

PhiSiGns provides flexibility to users in choosing the specific phage genomes of interest, BLAST E-value cut-off, BLAST alignment coverage cut-off, and primer parameter values. In addition, users can upload their own manually-curated alignments of selected signature genes to improve primer design. The results generated from each step of this tool are presented in a table, and can be downloaded to a local machine. BLASTP sequence similarity searches are pre-calculated (E-value = 10) for all phage genomes in the database, and can be parsed using different E-value and coverage cut-offs which considerably decreases the required computation time. The online version of PhiSiGns was developed to compare phages present in the existing database. For additional phage genomes, such as those that are not yet publicly available, running the PhiSiGns source code locally offers more flexibility and control.

PhiSiGns is the only tool currently available that combines the steps of signature gene identification with the ability to design PCR primers. Instead of requiring complicated user input files, since PhiSiGns is designed specifically for phage genomes, this program utilizes the phage genome and sequence annotation information from PhAnToME, which is available to users as the PhiSiGns local database. Thus no additional inputs (such as RefSeq IDs or sequence files) are needed from the user. Since primer design is an integral part of PhiSiGns, users do not need to worry about converting the output from the signature gene identification program into a format compatible with an existing primer design program. Compared to CODEHOP [46], Primaclade [66], and PriFi [67], PhiSiGns gives the user more flexibility in primer design parameters and is easier for phage biologists to use. CODEHOP [46] is one of the most commonly used programs for designing degenerate primers. Both PhiSiGns and CODEHOP utilize amino acid alignments; however, the downstream primer design process is significantly different in these two tools. From the amino acid alignment, CODEHOP produces a consensus amino acid sequence based on a position-weighted scoring matrix, and then creates a nucleotide consensus sequence based on the user-provided codon usage table. Therefore, some diversity may be lost through the CODEHOP primer design algorithm, since it is based on a dominant amino acid at each position and the relative codon frequency, as opposed to the actual nucleotide sequences present in the aligned genes. In contrast, PhiSiGns back-translates the amino acid sequences in the alignment into the original nucleotide sequences of the phage genes included in the analysis. Primers are then designed based on the consensus of this nucleotide sequence alignment, ensuring that all the genes included in the alignment will actually be recovered with the chosen primers. The output files generated by PhiSiGns are accessible in a simple text or tabular format, which can easily be used by other comparative genomic tools in further analyses. Compared to other available tools, PhiSiGns provides broad-range functionality in the primer design process to output all possibilities, from which the users can choose the best candidate primer pairs for their application. Overall, PhiSiGns is a simple and straightforward tool enabling comparative genomic analysis in the field of phage biology.

### Availability and requirements

**Project name**: PhiSiGns; **Project home page**: http://www.phantome.org/phisigns/; http://phisigns.sourceforge.net/; **Operating system**: Platform independent; **Programming language**: Perl; **Requirements for web-version**: Browser with JavaScript support; **Requirements for locally installed version**: Perl; BioPerl, BLAST, CLUSTALW; **Any restrictions to use by non-academics**: No

## Competing interests

The authors declare that they have no competing interests.

## Authors' contributions

BD designed the program, developed the standalone version, performed the phylogenetic analysis, and wrote the manuscript. RS developed the web-based version and helped design the program. DBG performed the PCR and cloning for the case study. RAE helped develop the phage database and coordinate with the PhAnToMe server. MB conceived the study, coordinated the work, and helped write the manuscript. All authors read, edited, and approved the final submitted manuscript.
